# The patterns of co-occurrence variation are explained by the low dependence of bark beetles (Coleoptera: Scolytinae and Platypodinae) on hosts along altitude gradients

**DOI:** 10.1186/s12983-022-00455-y

**Published:** 2022-03-04

**Authors:** Fang Luo, Ling-Zeng Meng, Jian Wang, Yan-Hong Liu

**Affiliations:** 1 College of Biological and Agricultural Sciences, Honghe University, Mengzi, 661199 Yunnan China; 2 Xishuangbanna Tropical Botanical Garden, Chinese Academy of Sciences, Mengla, 666303 Yunnan China

**Keywords:** Bark beetles, β-diversity, Spatial scales, Elevation gradient, Host dependence, Plant–insect interactions

## Abstract

**Background:**

Separation of biotic and abiotic impacts on species diversity distribution patterns across a significant climatic gradient is a challenge in the study of diversity maintenance mechanisms. The basic task is to reconcile scale-dependent effects of abiotic and biotic processes on species distribution models. Here, we used a hierarchical modeling method to detect the host specificities of bark beetles (Scolytinae and Platypodinae) with their dependent tree communities across a steep climatic gradient, which was embedded within a relatively homogenous spatial niche.

**Results:**

Species turnover of both trees and bark beetles have an opposite pattern along the climatic proxy (represented by the elevation gradients) at the regional scale, but not at local spatial scales. This pattern confirmed the hypothesis wherein emphasis was on influences of macro-climate on local biotic interactions between trees and hosted bark beetle communities, whereas local biotic relations, represented by host specificity dependence, were regionally conserved.

**Conclusions:**

At a confined spatial scale, cross-taxa comparisons of β-diversity highlighted the importance of simultaneous impacts from both extrinsic factors related to geography and environment, and intrinsic factors related to organism characteristics. The effects of tree abundance and phylogeny diversity on bark beetle diversity were, to a large extent, indirect, operating via changes in bark beetle abundance through spatial and temporal dynamics of resources distribution. Tree host dependence, which was considered and represented by host specificities, plays a minor role on the hosted beetle community in this concealed wood decomposing interacting system.

**Supplementary Information:**

The online version contains supplementary material available at 10.1186/s12983-022-00455-y.

## Background

Host specificity between a plant species and its hosted insects along environment gradients is a central issue in diversity maintenance. It is hypothesized that the distributional range of a hosted insect is largely determined by the ecological amplitudes of tree species with which they interact, if clear host specificity is observed [1]. That is, scarcity of suitable interacting partners can restrict the potential niche of a hosted insect species [2, 3]. This intrinsically dependent relationship has been used to estimate the global insect diversity through comparisons of trees or tree phylogeny diversity among different regions [4–8], and led to a recent controversy surrounding global estimates of insect species richness, ranging from 30 million [8] to 4–6 million [6]. However, some existed niche theories [9, 10] related to species distribution are based on spatially fine-grained variables associated with local biotic interactions and resource-consumer dynamics. This means that a potential bias should not be ignored when using the dependent plant-species diversity index as a surrogate to estimate regionally or globally hosted insect diversity.

The relative importance of biotic factors probably varies between regional and local interacting plants and insects communities because of the entanglements with abiotic factors [11]. Moreover, it depends on the spatial scale in which a compositional change is being considered [11, 12]. Thus, specific and well-designed sampling experiments are needed to overcome the interactions of different relative importance of biotic and abiotic factors among various spatial scales. A feasible approach to separate these two entangled factors from each other is to detect patterns of relationship between plants and insects beta diversity (hereafter ‘β-diversity’) [13], as species co-occurrence probably vary along a specific environmental gradient, at different spatial scales. Basic logic for this approach is that if distribution pattern was determined by insect host specificity, we can expect that plant and insect β-diversity will be correlated at local (fine), as well as regional (coarse) spatial scales. On the other hand, if patterns were resulted from parallel response to a broad abiotic environmental gradient, we will get a β-diversity distribution pattern only to be correlated at regional spatial scale [14].

**Fig. 1:**
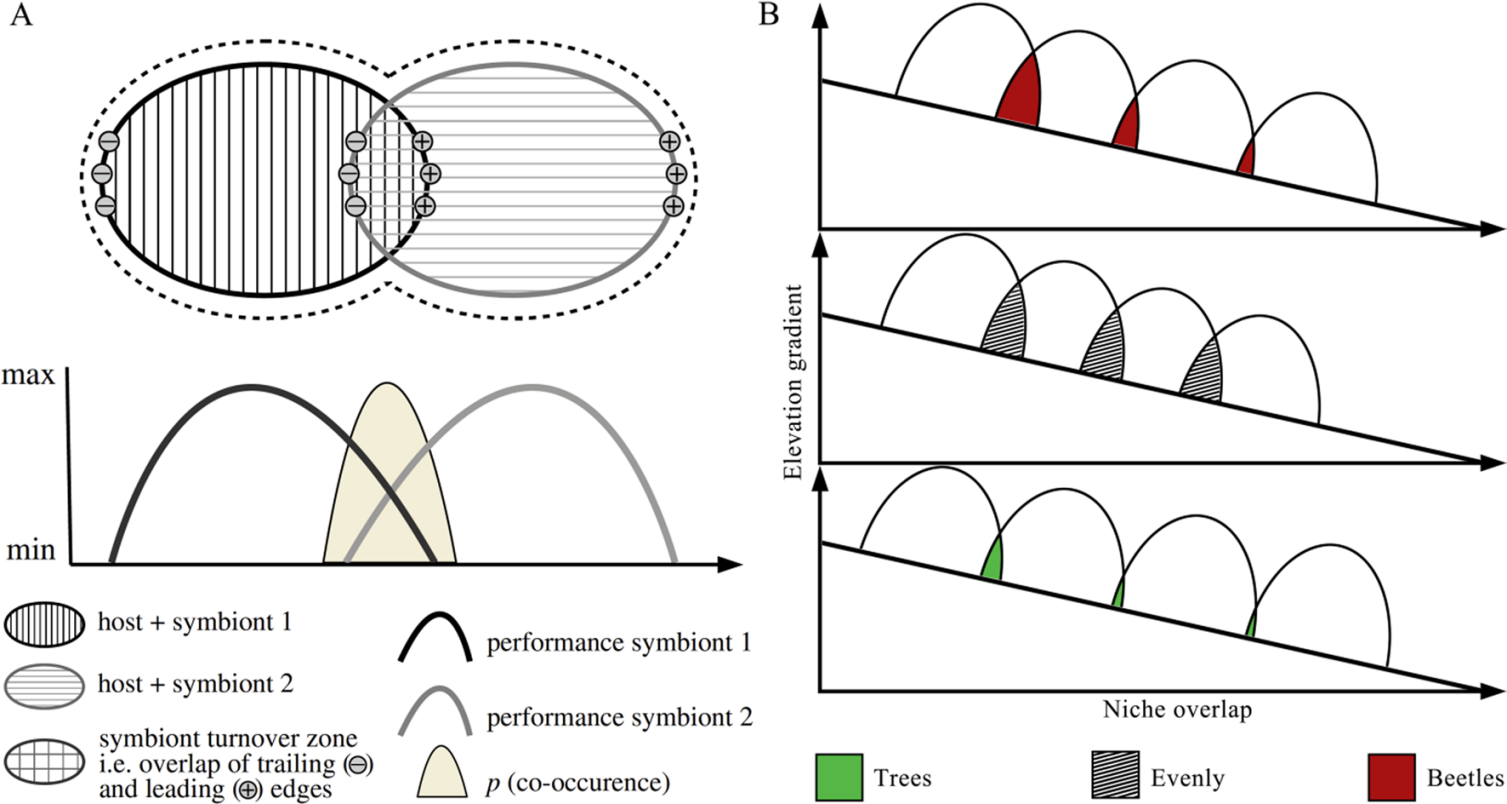
The space of a host can be subdivided into separate sections, each depending on the presence of particular symbionts (1 and 2) with distinct optimum performance along the respective niche axes (i.e., the elevation niche). Left panel **A** is re-sketched from the Fig. 1 of Rolshausen et al. (2020). Right panel **B** illustrates the niche overlap difference among different taxon across the same environmental elevation gradients

Spatial community structuring in host-specific niches implies the presence of demarcated transition zones where host replacement is most likely to occur (Fig. 1A). These ‘host turnover zones’ are derived from an integral but unspecified part of the mutualistic niche concept [15, 16]; it also can be calculated using β-diversity as aforementioned. Nevertheless, the niche overlap degree and turnover rate between neighboring spatial communities among different biological assemblages might vary (Fig. 1B), because of simultaneous impacts from extrinsic and intrinsic factors, related to geography and environment and to the evolutionary characteristics of organisms, respectively. At a global scale, the β-diversity of different communities generally decreases along the increasing latitude gradient (from tropical to temperate areas) [17–19]. Cross-taxon comparisons through meta-analyses showed a negative, albeit relatively weak, relationship between β-diversity and latitude [20, 21]. What's more interesting is that two β-diversity components, including nestedness and turnover which were already proposed by Baselga [22], had generally opposing patterns with regard to latitude at a global spatial scale [23]. Some evidences suggested that gradients in β-diversity occur as a result of latitudinal or elevation gradients and may be caused by a series of combined mechanisms. For example, deterministic processes of environmental or habitat filtering at a regional scale [24] and stochastic processes generating ecological drift at a local scale [25] impact spatial community assembly processes differently. Given the complexity of potential interactions among processes that are not mutually exclusive and are mediated by host specificity, it is not surprising that the relative importance of contributing factors and interactive mechanisms in driving patterns of β-diversity in hosted insects and plants remains largely unresolved.

Recent studies have attempted to overcome the spatial-scale dependence of diversity through a hierarchically nested method [11, 26, 27]. A hierarchical modeling framework [28] of β-diversity combined with phylogenetic and functional β-diversity was also proposed and may help to investigate mechanisms of local hosted community assembly. It suggests that if both host plant (henceforth referred as ‘host’) and insect turnover, which reflect bio-geographical impacts at all scales (from local to regional), occur simultaneously in response to geographic or environmental gradients, we would not expect to find any positive association between insect community composition and plant species/phylogenetic turnover after accounting for the influence of geography or the regional species pool. Therefore, if the observed insect turnover patterns were due to host specificity, a close relationship between insect and host species/phylogenetic diversity at both the fine and coarse spatial scales should exist. Comparatively, if parallel responses of both interacting assemblages to macro abiotic gradients were detected, we could conclude that the turnover patterns would be only correlated at large spatial scales. Separation of the impacts of macro climatic/environmental variables on community dissimilarities remains a challenge that has been rarely explored in the large-scale patterns of species richness. In the context of insect communities, we expect substantial compositional changes between regions (if historical bio-geographical effects are important) and along abiotic gradients within regions (if insects generally have specific abiotic requirements, such as temperature) [29, 30]. If insect speciation has been largely induced by allopatric host shifts of specialized insect species, we can expect a complete change in insect assemblage associated with shifts in plant assemblage [31, 32].

Many studies has mostly focused on plant-herbivore [6, 29, 33–35] and -pollinator interactions [36], relationships that are specialized more or less to some extent. Exceptions are Hulcr et al. [37], Hulcr et al. [3], Wu et al. [38], Wende et al. [39], and Vogel et al. [2], who studied detritivorous bark and saproxylic beetles, which are loosely associated with their hosts. Their studies investigated the impact of host specificity on beetle community composition within the same climatic region, disregarding environment gradients, and some found host specificities of plants played an important role on beetles’ composition [2, 39], some found not [3, 37]. Furthermore, the study aiming to explore the relationships between hosts and detritivorous saproxylic beetle community found that proportion of habitat specialists decreased, whereas proportion of habitat generalists increased with increased wood decay succession [38]. That is, the interactions of detritivorous insect assemblages with their hosts along steep environmental gradients have rarely been studied, and whether detritivorous insects exhibit similar trends to herbivores and pollinators with various host dependence extent is still unknown.

Bark beetles (Coleoptera: Curculionidae, Scolytinae, and Platypodinae) are the most important phloem-/wood-feeding guilds that threaten forests worldwide. There are two distinct groups of Scolytinaes, bark beetles and ambrosia beetles. Bark beetles are typically phloem feeders, while ambrosia beetles typically bore into the wood and feed on the symbiotic xylosaprophagous ambrosia fungi that the beetles introduce into the trees [40, 41]. Exploitation of the fungi as food source has allowed some ambrosia beetles to use a wide variety of hosts [37, 42]. The effects of tree diversity, host specificity, and structural heterogeneity of habitat on bark beetles at a local scale and along a flat environmental gradient have been well studied [2, 3, 37, 42–44]. The general goal of this paper was to derive environmental and spatial models to account for patterns of variation in the co-occurrence of bark beetles across a steep elevation gradient in the Yunnan Province, Southwest China. We aimed to compare the relationship between tree’s host specificity and bark beetle turnover among different climatic sub-regions embedded within a relatively homogeneous abiotic gradient. We hypothesized that divergent co-occurrence distribution patterns between host trees and bark beetle assemblages across huge environmental differences explain a weak relationship among these two groups, (2) trees’ host specificity plays a minor role in structuring bark beetle diversity distribution across regional elevation gradient compared with abiotic environmental factors, but not for local scale; and (3) β-diversity of both bark beetles and trees will be similar and there is a parallel spatial structuring of bark beetles with host trees along the steep elevation gradient, but with a much wider niche overlap compared with those host tree communities. Furthermore, we also predicted that β-diversity values of bark beetles will be significantly lower than their host trees across regional elevation gradient in the present study.

## Results

### Tree and beetle composition

A total of 64,710 bark beetles from 264 species were collected from all three sub-regions (see Additional file 2: Appendix file 1). The collection included 62,245 individuals representing 212 species of the Scolytinae group and 2465 individuals representing 52 species of the Platypodinae group. Of these, 25,676 individuals (180 species) were collected in tropical Bubeng; 33,781 individuals (116 species), in subtropical Ailaoshan; and 5,253 individuals (43 species), in cold temperate Lijiang. The five most abundant bark beetle species, which accounted for 51% of the total individuals collected were, in order of importance, *Scolytoplatypus raja*, *Scolytoplatypus blandfordi*, *Xylosandrus crassiusculus*, and two as yet unidentified species, *Microperus* sp. YUN08 and *Xyleborinus* sp. YUN02. A total of 2184 tree individuals from 213 species were recorded (see Additional file 3: Appendix file 2), including 1180 individuals from 137 species in tropical Bubeng, 795 individuals from 60 species in subtropical Ailaoshan, and 209 individuals from 18 species in temperate Yulongxueshan, Lijiang.

### Species turnover comparison between beetles and trees

**Fig. 2:**
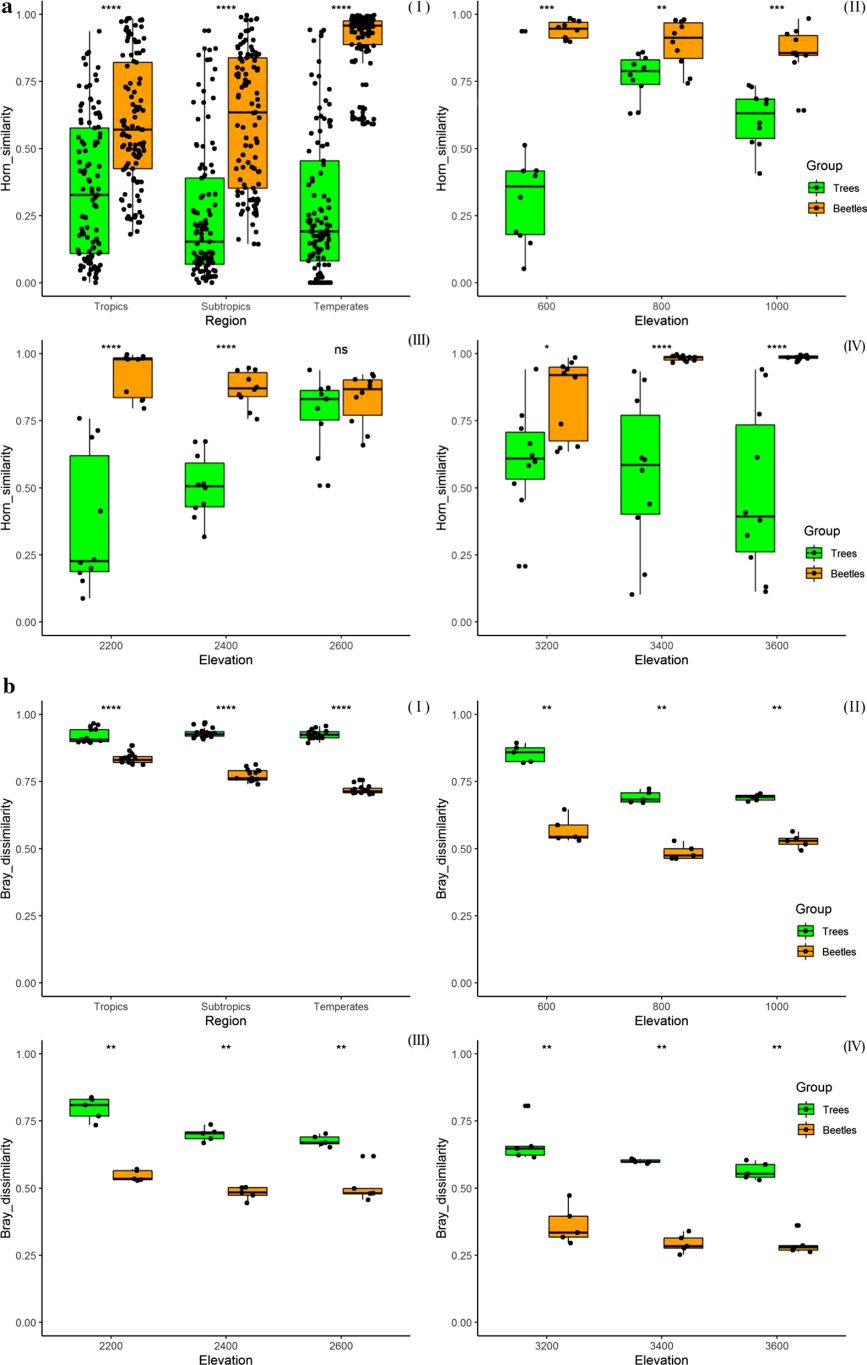

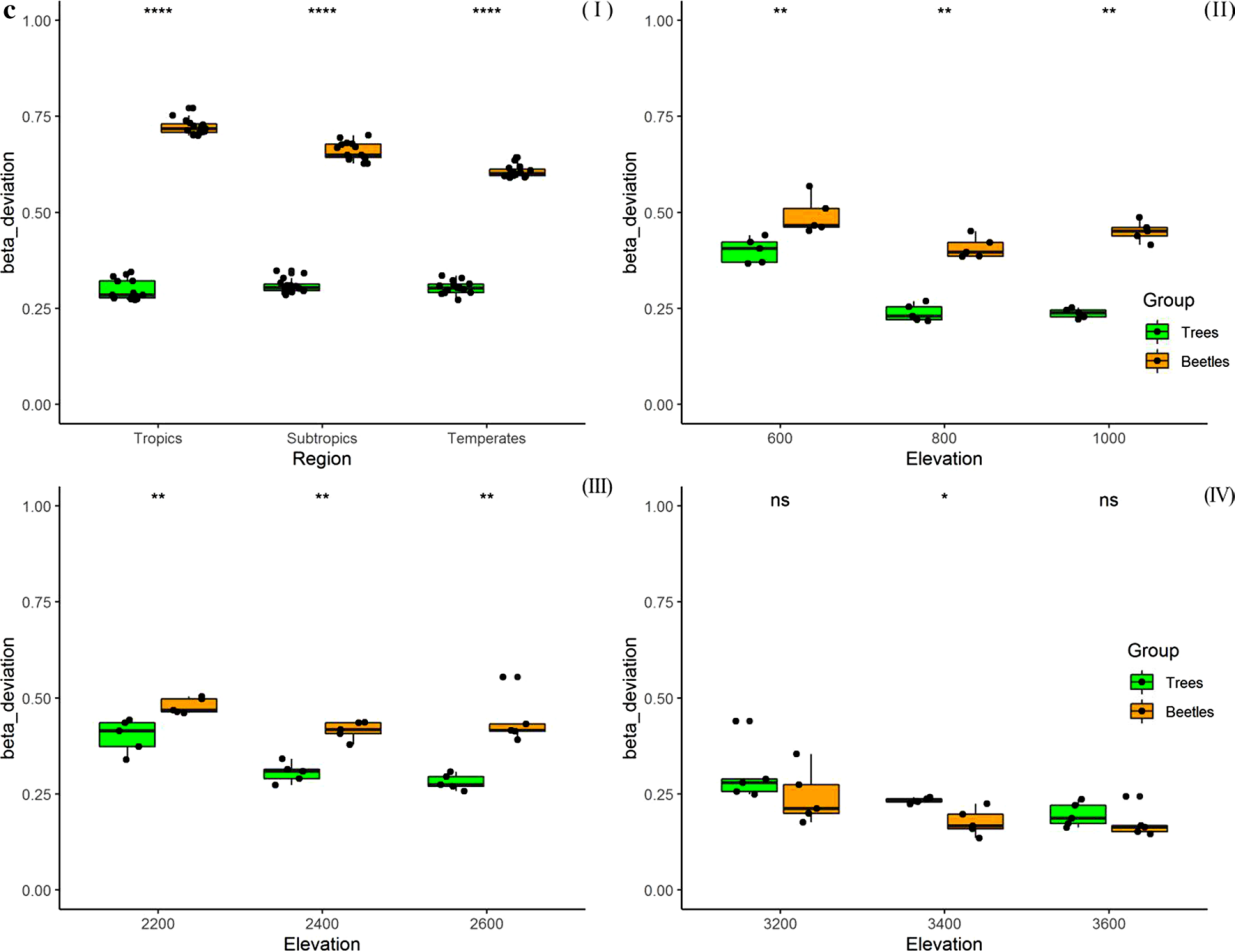
Turnover (Horn similarity) of insect species (bark beetles, yellow) and tree species (green) between Flight intercept trap sampling plots (25 × 20 m) at one regional scale Fig. 2a (I) and three local scales (II, III & IV) in Yunnan Province. Figure 2b shows the observed β-diversity calculated through Bray–Curtis dissimilarity method. Figure 2c shows the values of β-deviations through null model method which is based on the differences between observed β-diversity and a standardized effect size of β-diversity that controls sampling from the regional species pool. Boxes represent the median and 25th/75th percentile, and whiskers extend to 1.5 times the interquartile range

β-diversity of tree communities measured through Horn similarity method was significantly decreased with the elevation increased (GLMs, Z = − 5.905, *R*^2^ = 0.0376, *P* < 0.001) at the regional scale (Table 1) but with a very low *R*^2^ value. Contrary to this, β-diversity of beetles significantly increased with elevation gradient (GLMs, Z = 9.295, *R*^2^ = 0.2868, *P* < 0.001) (Table 1). Horn similarity β-diversity values of beetle assemblage were significantly higher than those of trees in three sub-regions, along the gradient from tropical to temperate (*P* < 0.0001; Fig. 2a-I). At the local scale, Horn β-diversity of trees and beetles also showed a significantly different pattern with elevation gradients in tropic, subtropics and temperate respectively. The opposite pattern at regional spatial scale was not similar with that showed in tropical Bubeng and subtropical Ailaoshan (Table 1). However, it was similar only for the upper altitudinal floor (Fig. 2a-IV), with a decreasing β-diversity of tree communities and increasing β-diversity of beetles when the elevation increased. Furthermore, most of the Horn similarity β-diversity values of beetle assemblages were statistically significant higher than those of trees communities (Fig. 2a-II, III & IV, *P* < 0.001), except for values at 2600 m in the subtropical areas (ns, Fig. 2a-III). When we compared the Horn similarity values of beetles and trees separately, they did not show a uniform tendency neither at regional scale, from tropical to temperate areas nor at local scale, from low to high elevation (Fig. 2a and Table 1).

**Table 1:** Results of Beta regression in Generalized linear models testing for the relationships between both beetles and trees composition variation at regional and local spatial scales with sampling elevation gradient respectively

Scales	Group	Horn_similarity			Bray_dissimilarity			Beta_deviation		
		*Z*	*R*^2^	*P*	*Z*	*R*^2^	*P*	*Z*	*R*^2^	*P*
Regional	Trees	− 5.905	0.038	< 0.001	0.477	0.004	0.634	0.94	0.020	0.347
*Yunnan*	Beetles	9.295	0.287	< 0.001	− 15.14	0.830	< 0.001	− 15.77	0.845	< 0.001
Local										
Tropics	Trees	2.430	0.157	0.015	− 5.341	0.684	< 0.001	− 6.184	0.700	< 0.001
* Bubeng*	Beetles	− 2.127	0.149	0.033	− 1.509	0.133	0.131	− 1.502	0.129	0.133
Subtropics	Trees	5.135	0.505	< 0.001	− 5.474	0.667	< 0.001	− 5.913	0.700	< 0.001
* Ailaoshan*	Beetles	− 3.326	0.277	< 0.001	− 1.406	0.117	0.160	− 1.404	0.115	0.160
Temperates	Trees	− 0.826	0.023	0.409	− 3.585	0.440	< 0.001	− 4.025	0.549	< 0.001
* Yulongxueshan*	Beetles	3.751	0.452	< 0.001	− 2.358	0.272	< 0.05	− 2.292	0.263	0.022

**Fig. 3:**
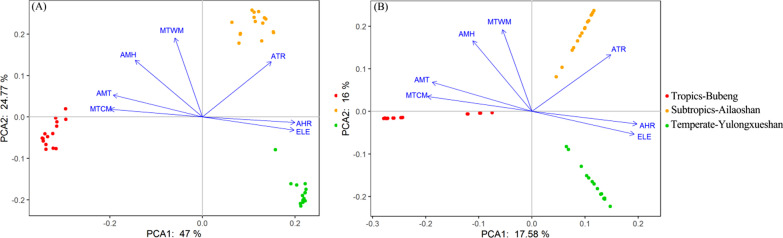
PCA analysis of the correlation between communities of bark beetles (**A**) and trees (**B**) with selected seven environmental variables at regional scale based on the RDA method. All bark beetles and trees abundances data were Hellinger transformed

The observed β-diversity values of tree assemblages measured through Bray dissimilarity method were significantly higher than those of beetles (*P* < 0.01; Fig. 2b). Almost both trees and beetles observed Bray β-diversity values showed a decreased tendency with elevation increased (Z < 0; Table 1) but except Z value of 0.477 for trees community at regional scale (Table 1). The *R*^2^ values of coefficients between both trees and beetles with elevation gradients also showed a statistically significant different pattern between regional and local spatial scales (Table 1). Bray dissimilarity β-diversity values of beetle assemblage were significantly lower than those of trees at regional scale, along the gradient from tropical to temperate (*P* < 0.0001; Fig. 2b-I). At the local scale, Bray β-diversity of both trees and beetles showed a decreased pattern with elevation increased (Z < 0; Table 1; Fig. 2b-II, III & IV). Nonetheless, it was not significantly for beetles in tropical Bubeng (*R*^2^ = 0.133, *P* = 0.131) and subtropical Ailaoshan (*R*^2^ = 0.117, *P* = 0.160). These patterns were not similar with Bray dissimilarity values of trees at regional scale and which showed a non-significantly increased pattern from tropics to subtropics and temperate (Z = 0.477; *P* = 0.634). The abundance β-null deviation measures showed an almost similar pattern with the β-diversity values through the method of Bray dissimilarity index calculation, and a large proportion of negative Z values means a decreased relationship with elevation gradient but except Z value of 0.94 for trees community at regional scale (Table 1; Fig. 2b and c). If we concentrated this point at local scales, β-null deviations of tree communities were significantly decreased with elevation gradient (*Z* < 0, *P* < 0.001; Table 1). Although β-null deviation of beetle assemblages were also decreased with elevation gradient (*Z* < 0; Table 1), it was not significantly correlated (*P* > 0.01; Table 1). The most of beetles β-null deviation values were statistically significant higher than those trees communities at both regional and local scales (*P* < 0.01; Fig. 2c-I, II & III) but except for those at 3200 and 3600 m plots in temperate areas (*ns*, Fig. 3c-IV).

### Correlations of beetle turnover with tree community

After removing the influence of spatial distance, RDA revealed that abundance of the 21 most important tree species of the 211 recorded explained 10% of the variation in wood-boring beetle community composition separately, spatial distance explained 1%, and 12% remained unexplained. Moreover, 14 phylogenetic PC axes (accounting for 90% of variation in tree phylogenetic turnover between sampling units) explained only 7% of the variation in bark beetle community composition separately. At the local scale, the selected tree species abundance after forward selection procedure in RDA analysis separately explained 34%, 3%, and 15% of beetles composition variation in the tropics, subtropics, and temperate area, respectively (Table 2).

**Table 2:** Results of redundancy analyses testing for the influence of tree species composition and phylogenetic turnover on the community composition of bark beetles in Yunnan Province, China, while removing the influence of spatial distance between sampling units (FITs)

		Number of species/phylogenetic PC axes included	*F*	*P*	Proportion explained
		Regional scale			
		Yunnan Province			
Beetles	Tree species	21/211	2.561	0.001	0.10
Beetles	Tree phylogenetic	14/45	2.275	0.001	0.07
		Local scale			
		Tropics (Bubeng)			
Beetles	Tree species	7/137	3.616	0.001	0.34
		Subtropics (Ailaoshan)			
Beetles	Tree species	4/60	1.258	0.188	0.03
		Temperates (Yulongxueshan)			
Beetles	Tree species	4/18	1.875	0.001	0.15

*F* statistics to test the significance of variables were calculated using the iterative ‘anova.cca’ in ‘vegan’. Regional spatial scale covers all of three local sampling sites from tropical Bubeng to subtropical Ailaoshan and to temperate Yulongxueshan

### Species distribution ordinations between beetles and trees

As shown in Fig. 3, PCA analyzes showed that all seven selected environmental variables have statistically significant cor-relationships with species distribution patterns for both trees and beetles communities (*P* < 0.05). Among them, the variables of elevation gradients (ELE) and relative annual humidity range (AHR) played a statistically significant negative role on species distribution. Considering even all environmental variables were spatial auto-correlated with elevation gradient, it’s not surprisingly that the minimum temperature of the coldest temperature (MTCM) have a positive impact on species distribution from temperate to tropical areas for both trees and beetles communities (*P* < 0.05, Fig. 3). More importantly, at the regional scales, the recorded environmental variables had a significantly higher explanation power for beetles than trees communities in the present study. It showed that two axis, represented by PCA1 and PCA1 reached to 47% and 24.77% separately for beetles group (Fig. 3A) but only 17.58% and 16% separately for trees (Fig. 3B).

## Discussion

Co-occurrences of bark beetles weighted as β-diversity did not all show a statistically significant correlation with both trees species and phylogenetic turnover at all three sub-regions from tropical to temperate areas. Those divergent co-occurrence distribution patterns between host trees and bark beetle assemblages across huge environmental differences explain a weak relationship among these two groups. This result suggests a minor role of host specificity on bark beetle diversity distribution at the community level. Several studies on comparison of insect host dependence among different climatic regions have presented diverse results. For example, a low host specificity of herbivorous insects in a tropical forest [6], species turnover of insect herbivore communities (leaf-chewing and sap-sucking guilds) in three Brazilian ecoregions was not related to tree richness [45], tree species had a lower impact on saproxylic beetle communities compared to abiotic factors (sun exposure) [2] and a greater turnover in caterpillar species composition in tropical faunas than in temperate faunas [46]. It is still reasonable to infer a relatively weak biotic interaction between trees and their hosted bark beetles when comparing the impacts of huge climatic differences from tropical to temperate climatic gradients. That is, elevation gradients, the most important environmental variable, are major drivers which structuralized the species distribution patterns at regional scale for both beetles and trees.

Admission of the minor role that insect-plant interactions play on large-scale hosted species turnover does not imply denial of the associations between different nested trophic assemblages. Tree host specificity was an important topic in biodiversity estimation and maintenance at local scale [8, 47, 48], although it is difficult to measure host specificities directly. First, comparing the strength of tree host specificities among different climatic areas generally ignore the impacts of the species pool and has led to diverse interpretations [6, 42, 46]. Second, simply using tree diversity as a surrogate to estimate their hosted herbivorous insect diversity [7, 8, 34, 37, 38, 49] is too coarse to get convincing unbiased results when considering the mixed effects of spatial environmental variables. Most of the different insect assemblages have shown a loose relationship with their hosts, and host shifting is a common phenomenon in nature [50]. If we further explore mutualistic interactions in detail, most of these interactions present a broad range of partners, even if it allows for ineffective partners to persist [15]. For bark beetles, as a major group of wood decomposers, a large part of this taxon has a strong dependence on their mutualistic fungus. It means external biotic selection pressure caused by the tree host specificity might be minor compared with other factors. Current studies suggest that extremely specialized or generalized insect species communities, whether detritivores or herbivores, only account for a minor proportion of the total assemblages, and most species are concentrated in the center group of the host dependence sequence [6, 7]. In this context, bark beetle diversity distribution was also probably rarely impacted by tree host specificities at the local spatial scale, especially when considering that limited host dependence will decrease quickly during the wood decomposition process [38].

Additionally, organism characteristics represented by dispersal type and ability play an important role on species diversity distribution across a large climatic gradient [21]. This dispersal ability, represented as access to dispersal or colonization over a relevant time interval, was another important component to mediate the actual spatial area of distribution of the species [51]. It is possibly that co-occurrences of interacted assemblages vary widely among organisms with different dispersal abilities [20, 23], when we did cross-taxon’s β-diversity comparison across a same environmental gradient. The present study showed a clear pattern that tree dispersal via seeds had a much higher turnover than the actively mobile bark beetles. It confirmed that species characteristics, reflecting the autecology of individual organisms, have a statistically significant effect on β-diversity distribution. Because the high dispersal ability significantly reduced the plot sampling difference, it should have a negative impact on β-diversity. Nonetheless, Harrison et al. [52] compared cross-taxon’ β-diversity with different dispersal ability for British biota ranging from flying birds to insects and plants. They showed that β-diversity is more constrained by species’ ecological niche requirements than by the species dispersal ability, and it could be negligible for statistical model comparison. The organisms in this study have huge differences in dispersal ability, and furthermore, the β-diversity of a specific biological assemblage might be controlled through a series of combined and interacting factors at different spatial and temporal scales. Considering that species dispersal ability can reflect species environment adaption flexibility to some extent [53], when species both adapt and disperse at the same time interval, dispersal and adaptation cannot combine positively to affect biodiversity maintenance [54]. Such conflicts can be observed and it gives us an idea that species diversity distribution is a paradox of mixed factors that cannot be easily and clearly separated. Although it is valuable to get a deep insight for comparisons of dispersal and adaptation among different biotic assemblage, our study presented a statistically significant different pattern of β-diversity between trees and their hosted bark beetles attributed most probably to those dispersal ability and type variation.

As we try to explore the major underlying mechanism that mediated the relationships between trees and the hosted bark beetles, many other technical factors, including effectiveness of sample size, spatial grain of sampling, bias of statistical method used, and data weight of each biological individual, can mix patterns that are not interpreted easily. Bennett and Gilbert [55] compared the differences between multi-site dissimilarity and traditional null model methods, and Engel et al. [56] used the coverage-based rarefaction method to overcome the species pool dependence of β-diversity bias toward to null hypothesis. No matter what kind of specific method is used nor how many effective samples were adopted, it is definitely a challenge to arrive at a convincing explanation based on statistical inference directly. Considering that although from a human perspective of the “local” scale, is standardized to be defined by 20 × 25 m plots in the present study, from the “point of view” of beetles and trees, these plots are not "equally local”, and the way these taxa perceive the environmental heterogeneity at this spatial scale could differ greatly just because of differences in their body size, which might impact on the human perception of their species turnover patterns. Furthermore, when we look back to compare the basic physiological differences between trees and beetles, tree distribution depends more heavily on soil water and nutrient content than do beetles. Both assemblages faced the same temperature gradient at a macro scale; bark beetles clearly have a broader low-temperature tolerance ability compared with that of tree communities because the majority of the beetle’s life cycle is confined to within the decaying tree stem. That is, if we did comparisons with some other insect assemblages which are not confined to wood boring ecological system, for examples, ants community [57] and herbivorous insects [58] in mountainous areas of Neotropical area, β-diversity of latter two groups, and which were mainly generated through turnover rate, were strongly influenced by variables correlated with elevation gradient, habitat structure and local resource distribution. From the data of species co-occurrence distribution in the present study, some of the beetle species collected in tropical Bubeng were also found in subtropical Ailaoshan, and some subtropical species were also occurred at temperate. It suggests that both host dependence and temperature gradient from tropical to subtropical and temperate areas do not have a statistically significant impact on bark beetle distribution. Thus, the parallel co-occurrence distribution pattern between bark beetle and tree assemblages at regional scale are most probably attributed to macro climatic gradient, and without relationships to trees host specificities clearly.

Finally, as previous study demonstrated that some bark beetle species rely on ethanol for host tree colonization because it promotes the growth of their fungal gardens while inhibiting the growth of “weedy” fungal competitors [59], it seems explained the most of bark beetle specimens were collected at the understory FITs in tropical and subtropical sampling areas. Because the tree stems at understory are generally relative bigger and older than canopies’ woods and can emit micro dose ethanol out from bark within dim light environment. The 75% alcohol used here might has influenced the community composition dramatically, making the ethanol-attracted component such as the genera of Scolytoplatypus and Xylosandrus are much more prevalent in our collections. As regarded to statistically non-significant differences of bark beetles collected between canopy and understory FITs in cold temperate, it was most probably attributed to the relative lower density of wood stems over there and similar light conditions among them.

## Conclusions

In this study, we compared co-variations in β-diversity of bark beetles and their host tree communities from the tropics to cold subalpine habitats in Yunnan, SW China. The results showed that species turnover of both trees and bark beetles have an opposite pattern along the climatic proxy (represented by the elevation gradients) at the regional scale, but not at local spatial scales. This pattern further supported the hypothesis according to which emphasis is on macro-climate influences on local biotic interactions between trees and hosted beetle communities, whereas local biotic relations represented by host specificities dependence are regionally conserved. At a confined spatial scale, cross-taxa comparisons of β-diversity highlighted the importance of simultaneous impacts from both extrinsic factors related to geography and environment, and intrinsic factors related to organism characteristics. The effects of diversity of tree abundance and phylogeny on bark beetle diversity were to a large extent indirect, operating via changes in beetle abundance through spatial–temporal dynamics of resource distribution. Tree host dependence, which was considered and represented by host specificities, plays a minor role on the hosted beetle community in this concealed wood decomposing interacting system.

## Methods

### Forest plots from tropics to temperate

**Fig. 4:**
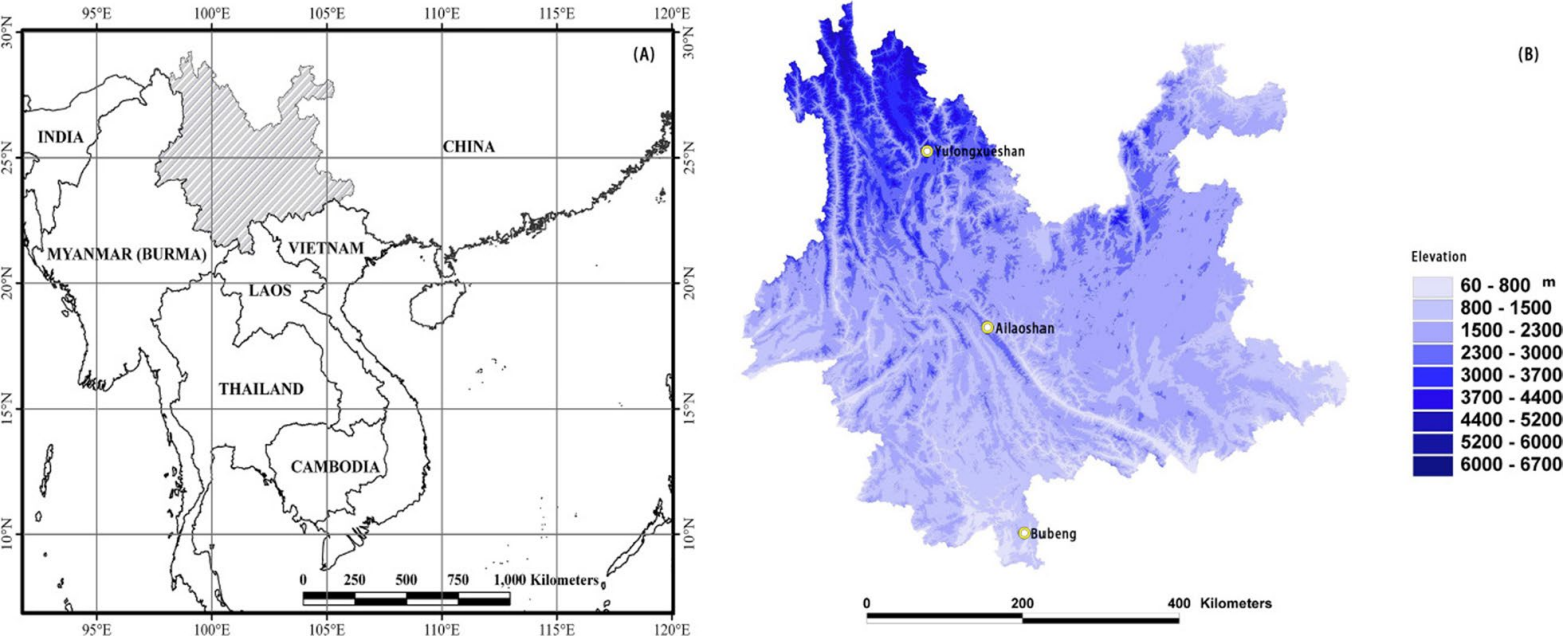
Left panel **A** shows the location of Yunnan (oblique dashed area), Southwest China. Right panel **B** shows the topography of Yunnan Province and the three sampling climatic sub-regions of the present study. Elevational transects were sampled in tropical (Bubeng; 600 m, 800 m, and 1000 m), subtropical (Ailaoshan; 2200 m, 2400 m and 2600 m), and subalpine (Yulongxueshan; 3200 m, 3400 m, and 3600 m) regions from low to high elevation. Map based mainly on the Figs. 1 and 4 of Zhu (2015)

The study was performed in the Yunnan Province, Southwest China, located centrally to the north of the Indo-China Peninsular (Fig. 4A). The area is extremely diversified in habitats and biodiversity, and it is one of the 25 biodiversity hotspots in the world [60]. The region sampled in this study is also part of the ‘Eastern Himalaya-SE Tibet hotspots’, and area with up to 3000–5000 or more vascular plants per 10,000 km^2^ [61, 62]. The impacts of the tropical monsoonal climate and complex mountainous topography cause this area to be covered with diversified vegetation, from tropical monsoonal rainforest to temperate coniferous forest, along elevation and altitudinal gradients.

We sampled bark beetles from three typical biomes: tropical monsoonal rainforest in Bubeng (Xishuangbanna); subtropical mid-mountain moist evergreen broad-leaved forest at Ailaoshan (Puer); and cold temperate spruce-fir forest at Yulongxueshan (Lijiang) from low to high elevation (Fig. 4B). Three big permanent plots for long-term ecological research with areas of 20 or 25 ha have been established in recent years, and a standardized vegetation inventory has shown that the area at Bubeng is hyper-diversified [468 woody species, [63]], the Ailaoshan plot is median-diversified [103 woody species, [64]], and the coniferous forest at Yulongxueshan is low-diversified [62 woody species, [65]]. Basic environmental information of each sampling plot are listed in Additional file 1: Table S1.

### Sampling design

A hierarchical and spatially nested sampling approach was used. To compare the impact of elevation on the β-diversity of bark beetle species across the three different sub-regions, we established three elevational transects in each sub-region, close to the permanent plots aforementioned. The experiment was performed from March 2018 to May 2019. In each area, we studied the same ecological amplitude of elevation gradient and covered a range of almost 400 elevation meters. Each transect included five forest plots with an intermediate cell size of 25 × 20 m, all of which were equipped with devices for collecting beetles. All five forest plots at each transect were oriented parallel to the respective contour lines and were at least 40 m apart. That is, each sub-region had fifteen cell sizes of 25 × 20 m plots and three sub-regions from tropical to temperate climate totally included 45 sampling plots of vegetation.

The tropical transects were located in Bubeng, Xishuangbanna (21°61′ N, 101°58′ E). This area borders Myanmar in the southwest and Laos in the southeast. Average annual temperature and precipitation are 22 °C and 1500 mm, respectively. The rainy season occurs from May to October and the dry season from November to April, with approximately 80% of the annual precipitation occurring in the rainy season. Heavy fog frequently occurs in the lowlands and valleys in the mornings during the dry season, and this fog expands the northern limit of the tropical rain forest from Southeast Asia. Three separated elevation transects (at 600, 800, and 1000 m) were established, based on vegetation and topography.

The subtropical transects were established in Ailaoshan, Puer (24°53′ N, 101°03′ E). Average annual temperature and precipitation are 11 °C and 1900 mm, respectively. The dry season occurs from December to April and the rainy season from June to October. The area encompasses a large tract of evergreen broad-leaved forests primarily dominated by *Lithocarpus* and *Castanopsis* species at mid-elevation (ca. 2200–2600 m a.s.l.) with a dense or sparse understory of bamboo and with *Rhododendron* dwarf forest toward higher elevations. Three separated elevation transects (at 2200, 2400, and 2600 m) were established based on their vegetation and topography.

The cold temperate transects were established at Yulongxueshan, Lijiang (27°14′ N, 100°23′ E). Average annual temperature and precipitation are 5.5 °C and 1600 mm, respectively. The rainy season occurs from July to September and the dry season from December to March of coming next year. The area encompasses a large tract of temperate coniferous forest with an understory formed primarily with *Berberidaceae*, *Caprifoliaceae*, and *Rosaceae* species. Trees of the families *Pinaceae* and *Fagaceae* dominate the canopy equally. Three separated elevation transects were established (at 3200, 3400, and 3600 m).

### Insect sampling and diversity estimation

Beetle sampling was performed using modified aerial collectors both in the canopy and understory of each forest plot at all sites. Aerial collectors, also called flight interception traps (FITs), were constructed with two hard transparent plastic plates (50 × 35 cm; H × W), which were arranged crosswise and fixed upon a plastic bowl (35 × 30 cm; D × H). A round plate of soft transparent plastic (45-cm diameter) was fixed over each FIT as a roof to prevent too much precipitation entering the trap during the rainy season. Within each plot, one trap was installed on tree branches in the canopy, at a height of 10–30 m above the ground, and a second was placed in the understory area at a height of 2 m. The FITs were fixed with nylon ropes to prevent the spillover of anti-rotting liquids by the wind. The collection bowls of the FITs were filled with a mixture of 75% alcohol and blue colored anti-freeze (ethylene–glycol) at a proportion of 1:2 v:v. Ten FITs were used in each transect, and a total of 90 FITs were installed across all three investigated regions.

Because of the large differences in climate among the three regions, the trap collection activity started at different times in each region; however, the sampling always covered the period of peak beetle activity and lasted at least one whole year. We conducted field work at Bubeng, from the beginning of April 2018 to the end of March 2019; at Ailaoshan, from the beginning of May 2018 to the end of April 2019; and at Yulongxueshan, from the beginning of June 2018 to the end of May 2019. At all plots, traps were emptied every 10 days during the collection period (with a few exceptions, where the traps were destroyed by strong winds or collection was impossible because of heavy rains). The beetle specimens were preserved in 70% ethanol and were preliminary sorted to morph-species level in the laboratory. Later all morph-species were further determined by Prof. Roger Beaver in Thailand and Dr. Heiko Gebhardt in Germany to the species level. Data analyses were based on numbers of species and individuals combined from all trap times per plot and the total counts from all collection periods. Voucher specimens of the collected beetles were temporarily stored in the laboratory at the Honghe University.

Both multi-sites dissimilarity and null model methods were used to calculate the β-diversity of beetle’s community. The first method used the package ‘vegetarian’ [66] in R 3.6.3 (R Core Team, 2013). The Horn similarity index was used as recommended by Jost [67], because it was the only overlap measure that was not disproportionately biased toward rare or common species. This index is considered to be a “true” measure that quantifies effective species overlap between sampling units [68]. The Horn similarity index was defined as: ^1^*D*_*β*_ = (ln2 – *H*_βShan_)/ln2, in which *H*_βShan_ is the Shannon entropy based on Hill numbers and β-diversity is therefore independent of α-diversity [67]. To overcome the demographic stochasticity of the sampling plots, a null model method was used to simulate species assemblages for each FIT by randomly sampling individuals from the regional species pool, according to the relative species abundance in the regional pool and the total number of individuals [69–71]. The dissimilarity matrix was calculated using the Bray–Curtis method, which takes account of species relative abundances [68]. From 1000 iterations of the null model, we calculated a standardized effect size (β-deviation) as the difference between the observed and mean expected dissimilarity, divided by the standard deviation of expected values. This formula retains the observed abundance distribution but randomizes the location of sampled individuals. The R code for β-null deviation calculations referenced the methods provided by Stegen et al. [72] and Tucker et al. [73].

The canopy and understory FITs were combined to give the smallest sampling unit for the estimation of β-diversity among three regions. Pairwise Horn similarities were calculated between all plots from beetle species abundance lists compiled for each sampling plot. The function ‘sim.table’ in ‘vegetarian’ was used for this purpose. Given that we were computing and comparing turnover between identical sampling units in all cases, it was not necessary to consider the species accumulation curve to assess whether sampling was adequate.

### Tree sampling and diversity estimation

The vegetation data collection was performed in April and May of 2019. The five 25 × 20 m forest plots established for the insect sampling at the three study regions were also used for the vegetation survey. Identical field methods were used to survey each plot. All plots were established as far away as possible from the large canopy gaps created by recent anthropogenic and natural disturbances. In each plot, we measured the abundance of each tree species (or morphospecies) ≥ 5.0 cm diameter at breast height (1.3 m). Seedlings were excluded. All sampling methods used in present study comply with the instructions of the Center for Tropical Forest Science (CTFS; http://www.ctfs.si.edu/) for collection of long-term, large-scale forest data from the tropics [76] and with those from the Chinese Forest Biodiversity Monitoring Network (http://www.cfbiodiv.org/). When establishing plots on slopes, we positioned the plot center line perpendicular to the slopes to minimize elevation gradients within the plots. As sampling included similar numbers of plots spanning small and large spatial distances, we were able to compare the potential influence of spatial limitation between regions at similar scales, including extents that encompass typical dispersal distances (seed shadows) from tropical to temperate vegetation.

Tree abundances were recorded for each plot. Horn similarity matrices were constructed using plots and transects as sampling units in the same manner as described for the beetles. The family and genus names of all the studied species (216 species in total) in the APG III system were obtained using the R package ‘plantlist’ [74], and their phylogenetic relationships were examined using the online Phylomatic tool ([75]; www.phylodiversity.net/phylomatic/) based on the angiosperm consensus tree from Davies et al. [76]. Similarity matrices were then constructed for plant phylogenetic β-diversity [PhyloSor Index [77]] with the function ‘phylosor’ in ‘picante’ package in R [78]. PhyloSor is a modification of the Sørensen similarity index that quantifies phylogenetic similarity of communities as the proportion of shared phylogenetic branch-lengths between two samples. If the length of shared branches is high, communities comprise phylogenetically closely related taxa.

### Environmental factors

We recorded air temperature and humidity data at each of the transects within the three study regions every 30 min using a thermo-logger (DS1923Hygrochron iButton®, Maxim, CA, USA) from April 2018 to May 2019, during the period of insect collection. A total of nine environmental data loggers were used; each device was fixed at one of the five canopy FITs in each transect. A total of seven variables were measured, including annual mean temperature (AMT) and humidity (AMH), annual temperature (ATR) and humidity ranges (AHR), maximum temperature of the warmest month (MTWM), minimum temperature of the coldest month (MTCM), and average elevation (ELE) of each transect. These data were assembled into a secondary environmental matrix and were prepared for canonical redundancy analysis (RDA). Detailed data are given in Additional file 1: Table S1.

### Data analysis

#### Spatial scale of species turnover

We first compared both beetle and tree composition turnover at local and regional scales using the multi-site dissimilarity method. A grouped plot-level similarity matrix was calculated and then partitioned into two different independent spatial components that reflected various β-diversity levels. It included turnover between sampling plots (total 15 plots) within three sub-regions and turnover between sampling plots among three sub-regions (total 45 plots). Considering all these turnover values produced by the multi-site dissimilarity method fall in the range from minimum zero to maximum one, beta regression method in Generalized linear models (GLMs) was performed to detect the relationships between both beetles and trees composition with variable of elevation gradient respectively. Then a Nonparametric Kruskal–Wallis ANOVA (analysis of variance) was further conducted to test for differences in Horn similarity values at various group and two spatial scales, followed by the appropriate post hoc tests. Data from the whole year of collections were combined for these analyses. The spatial component of turnover in tree species composition was investigated in an identical manner.

We also examined how β-null deviation values changed for meta-communities along the gradient from tropical to temperate for both tree and beetle community through beta regression method in Generalized linear models (GLMs) as mentioned above. The difference, in units of standard deviations, between the observed and mean expected raw turnover provided a measure of value that had sampling effects removed. The β-null deviation values based turnover estimates between tree and beetle assemblages are directly comparable to each other using a nonparametric Wilcoxon paired test, and any remaining correlation they had with gamma diversity (or other explanatory variables) could be interpreted as evidence for non-random ecological processes leading to intra-specific aggregation [79].

### Correlations of beetle turnover patterns

A correlation (RDA) approach was used to test for association of tree and beetle species composition while controlling for spatial distance between sampling plots. First, we used forward and backward selection in an RDA assessing the influence of the abundance of individual tree species (Hellinger transformed) on beetle species composition to identify the most important tree species for inclusion in the ordination. This was necessary because there were more tree species than sampling units in our dataset. The selection procedure was conducted using the ‘ordistep’ function in ‘vegan’ package. RDA (‘rda’ in ‘vegan’) was then performed on beetle species composition (Hellinger transformed) with the selected tree species as constraining variables and spatial distance [converted to a rectangular matrix using PCNM [80], the ‘pcnm’ function in ‘vegan’] as the conditioning variable (i.e., the effect which is removed). After that, variation partition was used to quantify the relative importance of spatial distance and trees abundance in determining the accompanying beetle species composition with an ‘anova.cca’ test in vegan. Similarly, to assess the impact of plant phylogenetic composition on beetle species composition, we first used phylogenetic principal components analysis (‘phyl.pca’ in ‘phytools’) to select the set of PC axes that explained 90% of variance in plant phylogenetic community composition. RDA was then performed on beetle species composition (Hellinger transformed) with selected PCNM converted spatial distance as the conditioning variable. *P*-values were assessed based on 999 random permutations. Because we wanted to explore the relationship between the dissimilarity of communities with environmental factors and spatial distance, we log-normalized the explanatory variables to make them comparable and then converted them to separated distance matrices. If both tree and beetle turnover occurred in response to climatic gradients or reflected bio-geographical influences (regional scale), we would not expect to find any positive association between beetle species composition and plant species/phylogenetic turnover after accounting for the influence of geography (local scale).

### Coordination of beetles and trees

To further compare the differences of relationship between beetles and trees with environmental variables at regional spatial scale, principal component analysis (PCA) was applied to the environmental variables, and the statistically significant components were selected by RDA. Beetle and tree species composition data were also Hellinger transformed. To quantify the homogeneity of dissimilarity variances within each transect, we compared the variances in the dissimilarity matrix using the ‘betadisper’ method [81]. This test is analogous to Levene’s test for homogeneity of ANOVA variances.

## Supplementary Information

**Additional file 1**. Table S1 showing the parameters of measured climatic variables of each FIT plot among three sample regions.

**Additional file 2**. Appendix file 1 showing a name list of total of 64,710 bark beetles from 264 species were collected from all three regions.

**Additional file 3**. Appendix file 2 showing a name list of a total of 2184 tree individuals from 213 species from all three regions.

## Data Availability

The dataset supporting the conclusions of this article are included within the article and its additional files.
