# Supplementary material for: The patterns of co-occurrence variation are explained by the low dependence of bark beetles (Coleoptera: Scolytinae and Platypodinae) on hosts along altitude gradients
Source: Front Zool. 2022 Mar 4;19:10. doi: 10.1186/s12983-022-00455-y (PMC8895613; doi:10.1186/s12983-022-00455-y)
Supplement: Supplementary file 1 — Additional file 1. Table S1 showing the parameters of measured climatic variables of each FIT plot among three sample regions. [file 12983_2022_455_MOESM1_ESM.docx]

Table S1 The parameters of measured climatic variables of each FIT plot among three sample regions.

|  | Annual  mean temperature  (°C) | | Annual temperature range  (°C) | Annual mean  Humidity (%) | Annual humidity range  (%) | Max temperature of warmest month  (°C) | Min temperature of coldest month  (°C) | Average  Elevation  (m) |
| --- | --- | --- | --- | --- | --- | --- | --- | --- |
| **Bubeng** | | |  |  |  |  |  |  |
| BB600 | | 19.37938 | 27.245 | 93.716613 | 75.19 | 27.42 | 9.32 | 705.2 |
| BB800 | | 21.45051 | 28 | 86.773607 | 81.2 | 35.5 | 8.5 | 838.2 |
| BB1000 | | 20.26883 | 23.5 | 87.333482 | 68.2 | 30 | 7.5 | 993.0 |
| **Ailaoshan** | | |  |  |  |  |  |  |
| ALS2200 | | 14.33506 | 40.968 | 85.655087 | 84.996 | 40.653 | -0.913 | 2368.2 |
| ALS2400 | | 13.00004 | 39.55 | 87.532566 | 91.187 | 34.637 | -0.922 | 2501.0 |
| ALS2600 | | 11.69748 | 41.564 | 89.456637 | 97.278 | 32.128 | -1.466 | 2692.2 |
| **Lijiang** | | |  |  |  |  |  |  |
| LJ3200 | | 8.928336 | 27.18 | 68.33484 | 98.325 | 26.117 | -1.327 | 3227.4 |
| LJ3400 | | 7.551954 | 30.29 | 73.91349 | 96.856 | 24.641 | -4.593 | 3332.6 |
| LJ3600 | | 6.162154 | 39.73 | 76.384518 | 94.258 | 21.616 | -7.497 | 3551.6 |
